# Uterine Polypi—I

**Published:** 1908-03-14

**Authors:** 


### GYNÆCOLOGY.

### UTERINE POLYPI.—I.

Polypoid growths of the uterus are of sufficiently common occurrence to make them a subject of general interest as regards diagnosis, prognosis, and treatment. The commonest variety of them is the mucous polypus of the cervix, whilst fibroid polypi occur less frequently, and placental or malignant forms least of all. It is true that placental remains are very often found in the uterus, but it is quite rare for them to assume a true polypoid form. Mucous polypi, however, are very common growths, and almost invariably occur as a result of chronic inflammatory changes in the cervical mucous membrane. Just as the so-called cervical erosion is a diffuse overgrowth of glandular structures lined by columnar epithelium, so the mucous polypus is a localised hypertrophy of the same nature. Its rosy red colour contrasting with the dull brownish pink of the vaginal aspect of the cervix makes it stand out very clearly. It may be only a small sessile growth as big as a pea, or it may be definitely stalked, and its size may increase up to that of a small hen's egg. The essential structure is that of an adenoma—that is to say it consists of a connective tissue stroma, more or less richly nucleated, containing branching gland tubules lined by long columnar epithelium. These have the characters of and are derived from the mucous glands of the cervical canal. The surface is covered, as a rule, by columnar epithelium, but it is quite common for this to be transformed into squamous stratified epithelium on those parts which are freely exposed in the vagina. As soon as the growths attain any size cystic changes nearly always occur in them: these are probably caused by inflammatory closure of the orifices of the gland tubules.

It is this cystic change which produces the large size these tumours sometimes attain. Polypi, having long pedicles, are liable to torsion and partial strangulation, so that they are easily infected, and may even become gangrenous and slough off. Acute torsion sometimes leads to breaking away of the growth without any gangrenous process. When small mucous polypi are very soft they may be overlooked when examination is made by the finger alone, a point which makes a visual examination imperative where symptoms point to some cervical lesion. At first only leucorrhœa is complained of, the source of the discharge being the cervical glands and those in the polypus itself. Sooner or later slight hæmorrhage, probably dependent upon partial strangulation, occurs, sufficient in amount to tinge the discharge with blood. This may be constant, or may only occur on coitus, defæcation or vaginal douching, though simple muscular exertion may be sufficient to cause it. Mucous polypi never affect menstruation, though there may be menorrhagia if the patient has endometritis in addition. It is this symptom which calls attention to something amiss, and, of course, carcinoma is suspected in many instances. The diagnosis from carcinoma gives but little difficulty, the points to remember being the localised pedunculated character of the growth, the soft velvety feel, and the absence of friability and induration at the base. The base may be hard, but it is the unyielding hardness of leather and not the friable, brittle hardness of cancerous induration. The cervix is nearly always very tough in these cases from chronic hyperplasia of the fibromuscular tissues.

To remove these polypi is simplicity itself. All that is necessary is to clean the vagina with antiseptic pledgets, and then to twist off the little mass with forceps. No anæsthetic is necessary, nothing more than slight oozing occurs after the operation, and no douching need be carried out. Probably if left alone mucous polypi would cause no more trouble than that of a constant discharge. They practically never become malignant, although, of course, a patient who has had one may acquire a malignant growth. It does not follow that the mucous polypus in any way predisposed to it.

Adenomatous polypi of this nature very seldom arise in the body of the uterus, except those of very small size, which are a part of one form of chronic hypertrophic endometritis. They have, however, been found, and can only be diagnosed after complete dilatation of the uterus, as their symptoms are the same as those of endometritis and carcinoma of the fundus. The other form of polypus of the cervix is a very rare one—the grape-like sarcoma. This is a malignant growth, and may occur at any age, having been seen in children as well as in adults. The symptoms of such a tumour are at first mucous discharge tinged with blood, or accompanied by more severe hæmorrhages, and later pain due to invasion of the uterus and surrounding structures. In appearance these are multilobular growths, semi-translucent and gelatinous to the sight, and very soft to the touch. They may grow to a large size, and completely fill the vagina or protrude from the vulva. Microscopically their appearances are very diverse. This is because these tumours are of a mixed character, suggesting an origin from undifferentiated mesoblast cells. The bulk of them is made up of a small round-celled growth like round-cell sacoma, and in addition they may contain striated and non-striated muscle, glandular structures, myxomatous tissue, and even cartilage. Their malignancy is very great, as they tend to infiltrate the uterus at the base, and then to invade the bladder or rectum. There might be a difficulty in distinguishing such a malignant growth from a mucous polypus in the early stages, but later their appearance is quite different, and microscopically at all times there is no difficulty whatever. As soon as the grape-like sarcoma is recognised a radical operation must be performed. Hysterectomy gives the only chance of ultimate recovery, and the histories of the published cases shows a very large percentage of recurrences and death.

